# Altered developmental neuroplasticity due to polysialyltransferase ST8SiaII deficiency in mice leads to schizophrenia-like phenotype

**DOI:** 10.1186/2193-1801-4-S1-L4

**Published:** 2015-06-12

**Authors:** Alexander Zharkovsky

**Affiliations:** University of Tartu, Estonia

**Keywords:** Neural cell adhesion molecule, sialyltransferase, neuroplasticity

Post-translational modification of the neural cell adhesion molecule (NCAM) by polysialic acid (PSA) is crucial for nervous system development and brain plasticity. PSA attachment is catalyzed by the two polysialyltransferases, ST8SiaII and ST8SiaIV. ST8SiaII dominates during embryonic and early postnatal development while ST8SiaIV is the prevailing enzyme of the juvenile and adult brain. A growing body of evidence links aberrant levels of NCAM and PSA to neuropsychiatric disorders, including schizophrenia. To investigate whether polysialyltransferase deficiency might cause a schizophrenia-like phenotype, ST8SiaII-/- mice, ST8SiaIV-/- mice and their wild-type littermates were assessed neuroanatomically and subjected to tests of cognition and sensory functions. ST8SiaII-/- but not ST8SiaIV-/- mice displayed enlarged lateral ventricles and a size reduction of the thalamus accompanied by a smaller internal capsule and a highly disorganized pattern of thalamocortical and corticothalamic fibers. Loss of ST8SiaII was associated with reduced phosphorylation of fibroblast growth factor receptor 1 in the frontal cortex of newborn mice and retarded neuronal differentiation of newly generated cells in the dentate gyrus of adults. ST8SiaII-/- and ST8SiaIV-/- mice were both impaired in short- and long-term recognition memory, but only ST8SiaII-/-mice displayed impaired working memory and a deficit in prepulse inhibition, which could be attenuated by clozapine treatment. Furthermore, ST8SiaII-/- mice exhibited anhedonic behavior and increased sensitivity to amphetamine-induced hyperlocomotion. These data reveal that reduced polysialylation in ST8SiaII-/- mice leads to pathological brain development and schizophrenia-like behavior. We therefore propose that ST8SiaII deficiency has the potential to cause a neurodevelopmental predisposition to schizophrenia.

